# NCX 1000 Alone or in Combination with Vitamin E Reverses Experimental Nonalcoholic Steatohepatitis in the Rat Similarly to UDCA

**DOI:** 10.4061/2011/136816

**Published:** 2011-10-16

**Authors:** Yara Haddad, Diane Vallerand, Antoine Brault, Jean Spénard, Pierre S. Haddad

**Affiliations:** ^1^Natural Health Products and Metabolic Diseases Laboratory, Department of Pharmacology Université de Montréal and Montreal Diabetes Research Center, Montreal, QC, Canada H3C 3J7; ^2^Institute of Nutraceutical and Functional Foods, Laval University, Sillery, QC, Canada GIV 0A6; ^3^Department of Pharmacology, Université de Montréal, Montreal, QC, Canada H3C 3J7; ^4^R & D Axcan Pharma Inc, Mont-St-Hilaire, QC, Canada J3H 6C4

## Abstract

We explored the therapeutic effect of NCX 1000, a derivative of ursodeoxycholic acid (UDCA) with nitric oxide (NO) donating properties, alone or in combination with vitamin E, in an experimental model of NASH in the rat. *Methods*. A control group was fed a standard liquid diet (Control), and the NASH groups were fed a high-fat liquid diet for 12 weeks without (NASH) or with simultaneous daily gavage with either NCX 1000 at 15 or 30 mg/kg (N15 and N30, resp.), or N15 plus vitamin E 100 mg/kg (N15  + VitE) for the last 6 weeks; UDCA 17.2 mg/kg was used as a reference. *Results*. NASH rats developed all key features of the disease. Treatments with N30 improved liver histology, decreased lipid peroxidation, and completely suppressed increases in LDH release, plasma insulin, and TNF-*α*. It also decreased O_2_^∙−^ release and returned liver weight and glutathione back to normal. All effects were similar to the reference treatment, UDCA. The N15 treatment was less efficient than the N30 group, but became comparable to the latter when combined to vitamin E. *Conclusion*. Our study demonstrates that NCX 1000 has potent cytoprotective, antioxidant, and hypoinsulinemic properties that can be enhanced by combination with vitamin E.

## 1. Introduction

Nonalcoholic steatohepatitis (NASH) was characterized for the first time by Ludwig and colleagues in 1980 [1] as the inflammatory stage following reversible steatosis in the liver. It is part of the nonalcoholic fatty liver disease (NAFLD) spectrum, which is now the most common cause of hepatic illness. NASH is more likely to develop in sedentary inactive people, following high-fat/high-caloric diets, notably in possible association with visceral adiposity. It is strongly related to the metabolic syndrome, being its hepatic expression, and to type II diabetes mellitus [2–5]. Although the pathogenesis of NASH is not entirely elucidated, it is now well established that it involves a multiple-hit process [6]. This includes insulin resistance and oxidative stress and underlies the development of macrovesicular steatosis, necroinflammatory lesions, Mallory body formation, hepatocyte ballooning, and collagen deposition [7–10]. Furthermore, elevated inflammatory cytokines, elevated or decreased adipokines, increased lipid peroxidation products, and increased reactive oxygen species play a major role in the pathogenesis of NASH [6, 11]. Several remedies were investigated for the treatment of NASH but an efficient therapy has yet to be developed. 

Ursodeoxycholic acid (UDCA) is a hydrophilic biliary acid known to possess antioxidant properties [12–14]. Its cytoprotective, antiapoptotic, and membrane stabilizing and immunomodulative effects have also been acknowledged by Angulo [15]. It is presently approved for the management of primary biliary cirrhosis and other cholestatic and noncholestatic liver diseases [16–18]. Several clinical studies have shown that treating NASH patients with UDCA improved aminotransferase levels [19–21], adiponectin levels [19], and grade of steatosis [20, 22]. UDCA was also found effective in enhancing the therapeutic effects of low caloric diet to prevent fat-induced NASH in the rat [23]. However, its efficiency was challenged when it failed to show improvement in transaminase levels and in steatosis beyond that found in the placebo group in a doubled-blind random controlled study [24].

NCX 1000 is a derivative of UDCA with nitric oxide (NO) donating activity that was reported to be efficacious in animal models of liver injury due to its immunomodulatory, cytoprotective and antiapoptotic properties [25–30]. Results from our previous study show that NCX 1000 exerts a better protective effect than UDCA on mouse hepatocytes treated with amiodarone to produce NASH-like lesions *in vitro* [31]. Moreover, additive protection was observed with a combination of NCX 1000 and lipophilic antioxidants such as vitamin E. However, no animal or clinical studies have yet assessed the potential benefit of NCX 1000 for the treatment of NASH.

Thus, based on the previous encouraging *in vitro* results, we tested the beneficial effects of NCX 1000 in an experimental model of NASH in the rat and used UDCA at an equimolar high dose as comparator. We also assessed the effect of combining a lower dose of NCX 1000 with vitamin E and evaluated the impact of all treatments on oxidative stress, inflammation, and the metabolic syndrome parameters.

## 2. Materials and Methods

### 2.1. Chemicals

All chemicals were purchased from Sigma-Aldrich (Oakville, ON, Canada). UDCA and NCX 1000 were graciously provided by Axcan Pharma Inc. (Mont-St-Hilaire, QC, Canada).

### 2.2. Animals and Experimental Protocol

Male Sprague-Dawley rats (75–100 g) were purchased from Charles River Canada (St-Constant, QC, Canada). They had access to normal rat chow diet and water for 5 days before the beginning of experimental protocols. They were then randomly double-housed and divided into 2 groups of 6 rats and 4 groups of 10 rats. The control group (Control, *n* = 6) was fed the Lieber-DeCarli standard liquid diet (Dyets Inc., Bethlehem, PA) for 12 weeks. The nonalcoholic steatohepatitis group (NASH, *n* = 6) received the Lieber-DeCarli high-fat liquid diet (Dyets Inc., Bethlehem, PA) for 12 weeks. The treatment groups (*n* = 10, each) were fed the same high-fat liquid diet for 12 weeks to which was added the following respective treatments during the last 6 weeks; NCX 1000 30 mg/kg (N30), NCX 1000 15 mg/kg (N15), NCX 1000 15 mg/kg combined with vitamin E 100 mg/kg (N15 + VitE), and the reference treatment group UDCA 17.2 mg/kg (UDCA), daily by intragastric gavage in a 0.5% methylcellulose solution. The UDCA dose of 17.2 mg/kg corresponds to the upper normal therapeutic range for that drug in clinical studies. In term of UDCA content, it is equivalent to that of 30 mg/kg of NCX 1000. Diets were prepared freshly on a daily basis. The Lieber-DeCarli standard liquid diet contains 35% of energy derived from fat, 18% from protein, and 47% from carbohydrates. The fat content of this diet is equivalent to that found in an average normal US diet and is classified as “healthy” by the Institute of Medicine, according to Lieber et al., 2004 [32]. In contrast, the high-fat liquid diet contains 71% of energy derived from fat, 18% from protein, and 11% from carbohydrates [32]. 

The weight of animals and their food consumption was monitored weekly. All animals were fed the diets *ad libitum* and kept with controlled temperature and humidity, under a 12 : 12-hr light-dark cycle. All experiments were performed in accordance to the guidelines of the Canadian Council on the Care of Animals and approved by the University's Ethics Committee.

### 2.3. Liver Isolation

Overnight fasting rats underwent laparotomy after they were anesthetized with sodium pentobarbital (50 mg/kg b.w., intraperitoneally) and the portal vein was cannulated following the procedure described by Huet et al., 2004 [33]. Blood was collected via the inferior vena cava and blood glucose was directly evaluated using a commercial glucometer (One Touch Ultra, LifeScan, Johnson & Johnson, Milpitas, CA). Livers were flushed with Krebs-Henseleit (NaCl 0.9%, KCl 4.8 mM, MgSO_4_ 1.2 mM, NaHCO_3_ 25 mM, CaCl_2_ 2.1 mM, pH 7.4, 22°C) buffer for 3 minutes then removed and weighed. Thereafter, liver dissection was performed. Pieces from the median lobe were placed in a 10% formalin solution for histopathological analysis. Other tissue samples were used fresh for biological determinations (see below) or immediately frozen in liquid nitrogen then stored at −80°C until use.

### 2.4. Histology

Histological sections and hematoxylin phloxine saffron staining (HPS) were done by the Department of Histology of St-Justine's Children Hospital (Montreal, QC, Canada). Each section was scored for steatosis based on the percentage of hepatocytes containing macrovesicular fat and according to published criteria (grade 0: absent; grade 1: less than 33%; grade 2: 33–66%; grade 3: more than 66%) [34, 35]. Inflammation was expressed as the mean of cell counts in 10 randomly selected areas (at 400x) within each slide using the following scale (0: normal; 1: mild; 2: moderate; 3: severe) [35]. We evaluated Mallory bodies and collagen deposits (using HPS staining) as present or absent. Pictures were taken using Axio-imager electronic microscope (z1 Zeiss, Jena, Germany) and Axio-vision 4.2 software.

### 2.5. Blood Parameters

Plasma transaminases (ALT and AST) levels, lactate dehydrogenase (LDH), triglycerides (TG), total cholesterol (TC), low-density lipoprotein (LDL), and high-density lipoprotein (HDL) were measured by the Department of Biochemistry of St-Justine's Children Hospital (Montreal, QC, Canada). Plasma tumor necrosis factor-*α* (TNF-*α*) was assessed using Quantikine Rat TNF-*α* Immunoassay kit (R&D Systems, MN, USA). Serum adiponectin measurements were performed using Rat Adiponectin ELISA Kit (AdipoGen, Seoul, Korea). Plasma insulin was evaluated with the Rat Insulin Radioimmunoassay (RIA) kit (Linco Research, St. Charles, MO). The homeostasis model assessment for insulin resistance (HOMA-IR) index was calculated using insulin and glucose values with the following formula: (fasting insulin (mU/L) × fasting blood glucose (mmol/L)/22.5) [36]. 

### 2.6. Mitochondria Isolation

Mitochondria were isolated from 1 g of fresh liver as described by Johnson and Lardy [37]. Briefly, tissue was homogenized on ice using a Teflon potter homogenizer in the isolation medium containing sucrose (250 mM), Tris-base (10 mM), EGTA (1 mM), and pH 7.2 at 4°C. To remove cellular fragments, homogenate was centrifuged at 600 g for 10 min at 4°C. Supernatant was collected and centrifuged at 15000 g for 5 min at 4°C. The pellet was then softly washed once with the same medium, centrifuged, and washed another time with the isolation EGTA-free medium and centrifuged again. The final pellet, containing viable mitochondria, was suspended in this last medium and stored on ice. Protein content was determined according to the Lowry method [38].

### 2.7. Oxidative Stress Assessment

Liver lipid peroxidation was determined by measuring the MDA levels, applying the thiobarbituric acid (TBA) method, as described by Ligeret et al. [39] with a few modifications. Briefly, fresh liver (0.5 g) was homogenized on ice using a Teflon potter homogenizer and a polytron in a sucrose buffer (4.5 mL, 250 mM). Homogenate was centrifuged at 2000 g for 30 min at 4°C. The supernatant was collected and protein content was determined using the Bradford method. Supernatant (200 *μ*L) was added to a vial containing an 8.1% (w/v) sodium dodecyl sulfate solution, a 20% (v/v) acetic acid solution (pH 3.5), a 0.8% (w/v) TBA solution, and distilled water. Vials were heated at 95°C for 45 min and cooled on ice for 2 min. After butanol (4 mL) was added, all vials were mixed for 15 min and centrifuged at 1000 g for 10 min. Supernatants (200 *μ*L) were transferred into a 96-well plate and thiobarbituric acid reactive substances (TBARS) were estimated by measuring fluorescence (*λ*_ex_ = 530 nm; *λ*_em_ = 590 nm) using a multilabel counter model Wallac Victor^2^ (Perkin Elmer, Woodbridge, ON). The data were compared to a standard curve of MDA (0 to 84 *μ*M) and results were expressed as nmol MDA/mg of protein. 

Lucigenin-amplified chemiluminescence was used to determine superoxide anion in liver tissue following the procedure described by Oliveira and colleagues [40]. Briefly, a frozen liver fragment was incubated at 37°C for 15 min in an oxygenated (95% O_2_-5% CO_2_) Krebs-HEPES buffer containing NaCl (118.3 mM), KCl (4.69 mM), CaCl_2_ (1.87 mM), MgSO_4_ (1.2 mM), KH_2_PO_4_ (1.03 mM), NaHCO_3_ (25 mM), glucose (11.1 mM), and Na-HEPES (20 mM), pH 7.4. The fragment was then put into a scintillation vial containing 2 mL of this buffer, supplemented with lucigenin (250 mM). A scintillation counter (Wallac 1409 Model, Perkin Elmer life science, St-Laurent, QC), adjusted for single-photon emission recording mode, was used for counts appraisal during a 15 min period. Data were obtained from the area under the counts versus time curve and expressed as a function of the dry tissue weight (mg).

### 2.8. Reduced Glutathione

Fresh liver (0.5 g) was minced and homogenized on ice using a polytron in 1 mL of a metaphosphoric acid solution (5%) prepared daily. Homogenate was centrifuged at 3000 g for 10 min at 4°C. Supernatant was used for reduced glutathione (GSH) measurement using the Bioxytech GSH-400 Assay Kit Oxis International inc, Portland, OR) according to the manufacturer's instructions. Absorbance at 400 nm was assessed using an Ultrospec 2100 pro spectrophotometer (Biochrom, Cambridge, England). The data were compared to a standard curve of reduced GSH (0–90 *μ*M).

### 2.9. Mitochondrial ATP

Freshly isolated mitochondria (100 *μ*L) were added to ice-cold HClO_4_ (900 *μ*L, 1M) [38]. Centrifugation was done at 2000 g for 10 min at 4°C and supernatant (100 *μ*L) was neutralized with KOH (47 *μ*L, 2 M) and Tris-HCl (853 *μ*L, 100 mM) [39]. Measurement of bioluminescence was carried out with the ATP Bioluminescent Assay Kit (SIGMA-Aldrich, Oakville, ON) according to the manufacturer's instructions using a multilabel counter model Wallac Victor^2^(Perkin Elmer, Woodbridge, ON). The data were compared to a standard curve of ATP from 0 to 80 *μ*M and results were expressed in *μ*mol ATP/g of protein.

### 2.10. Western Blot Analysis

Western blots on total liver homogenate were performed with a rabbit polyclonal anti-PPAR-*α* primary antibody (1 : 400) (Santa Cruz Biotechnology Inc. Santa Cruz, CA) similarly to procedures described by Ouazzani-Chahdi et al. [31]. Liver microsomal fraction was also used in western blot with mouse monoclonal anti-rat P450 CYP2E1 antibody (1 : 1000) (Oxford Biomedical Research, Oxford, MI). Secondary antibodies used are the following: a goat polyclonal anti-rabbit (1 : 100 000) (Jackson Immunoresearch Laboratories, Baltimore, PA) and a goat anti-mouse (1 : 4000) (Cell Signaling Technology, Danvers, MA). Immunoreactive proteins were detected using enhanced chemiluminescence system following the manufacturer's instructions GE Healthcare (ECL Western Blotting Detection Reagents, Buckinghamshire, UK). Bands quantification was achieved using ImageJ 1.37 v (NIH, USA).

### 2.11. Statistical Analysis

All results were expressed as means ± SEM. Group means were compared by one way ANOVA followed by Fisher's PLSD test (and the Chi square test for histological contingency table) using StatView 5.0.1 (SAS Institute Inc. NC). A *P* value < 0.05 was considered statistically significant.

## 3. Results

### 3.1. Changes in Body Weight and Liver Weight

After 12 weeks of the NASH-inducing fat diet, there were no significant differences in body weight (BW) or weight gain (WG) between groups. The liver weight (LW) decreased significantly in the N30 group compared with the NASH group (*P* < 0.05), similarly to the UDCA group. Furthermore, liver index (LW/BW × 100%) increased very significantly in the NASH group as compared to the control group and decreased significantly in all treatment groups (*P* < 0.05) (Table 1).

Measurements were obtained from rats on the day of sacrifice, after 12 weeks of standard liquid diet (Control) or high-fat liquid diet alone (NASH), or with treatment for the last 6 weeks with UDCA (17.2 mg/kg), NCX 1000 (30 or 15 mg/kg) (N30, N15 resp.), or NCX 1000 plus vitamin E (15 mg/kg and 100 mg/kg, resp.) (N15 + VitE). Body weight (BW), weight gain (WG), liver weight (LW), and liver index (LI = LW/BW × 100%). Values are expressed as means ± SEM of 6–10 rats. *P < 0.05 versus control group. ^†^ *P* < 0.05 versus NASH group.

### 3.2. Glycemic Homeostasis

**Figure 1:**
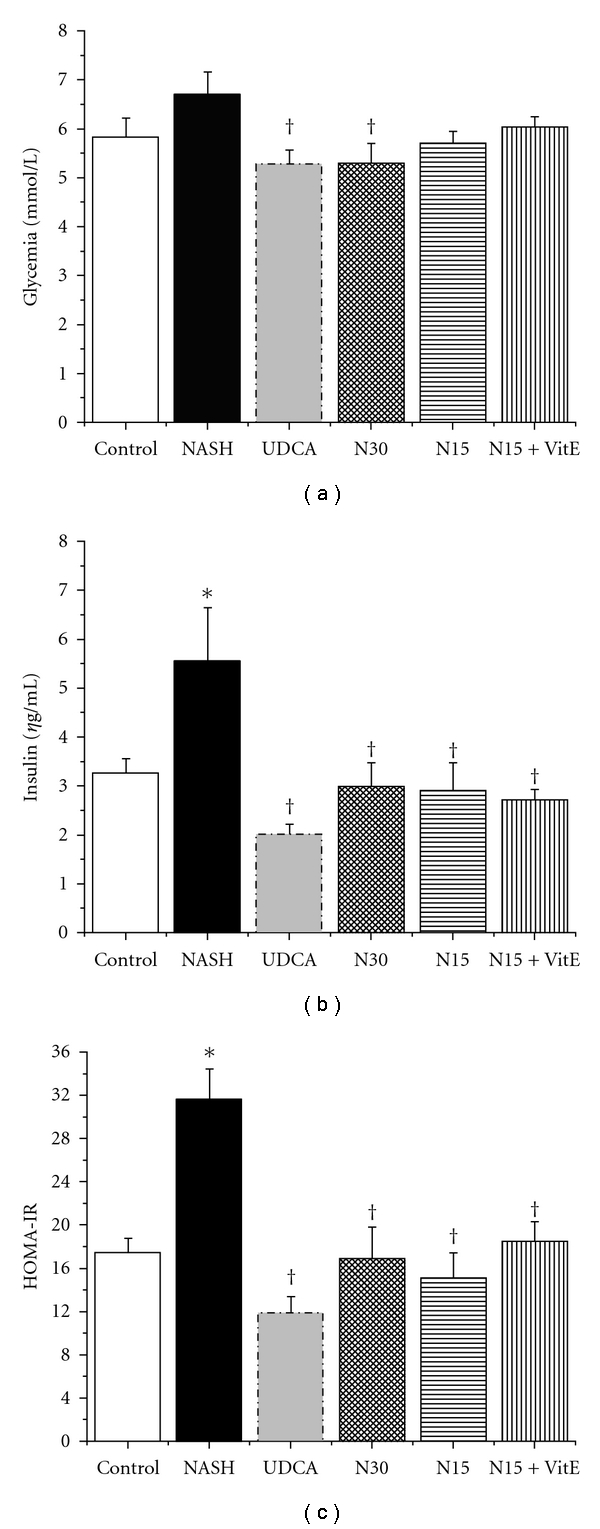
Effect of treatments on glycemic homeostasis.

Fasting glycemia in the NASH group tended to be slightly higher than that of the control group, but this effect failed to reach statistical significance. However, N30 treatment significantly lowered plasma glucose as compared to the NASH group, a property similar to that of the UDCA reference treatment (Figure 1(a), *P* < 0.05). In contrast, the NASH treatment caused a frank hyperinsulinemic state as compared to the control group (plasma insulin values of 5.56 ± 1.09 and 3.26 ± 0.31 *η*g/mL, resp; Figure 1(b), *P* < 0.05). This situation was associated with a substantial increase in insulin resistance, as demonstrated by the significantly greater value of HOMA-IR in NASH animals as compared to control congeners (Figure 1(c), *P* < 0.05). Both plasma insulin and HOMA-IR values were decreased significantly by all drug intervention groups as compared to NASH treatment (Figures 1(b) and 1(c), *P* < 0.05). Indeed, values in N30, N15, and N15 + VitE groups were not statistically different from each other as well as from control animals or from UDCA reference group. 

Glycemia Figure 1(a) was measured in whole blood and insulin, Figure 1(b) was measured in plasma obtained from rats in the fasting state, after 12 weeks of standard liquid diet (Control), high-fat liquid diet alone (NASH), or with treatment for the last 6 weeks with UDCA (17.2 mg/kg), NCX 1000 (30 or 15 mg/kg) (N30, N15 resp.), or NCX 1000 plus vitamin E (15 mg/kg + 100 mg/kg) (N15 + VitE). HOMA-IR, Figure 1(c) is indicative of the insulin resistance state for the animals. Values are expressed as means ± SEM of 6–10 rats. *P < 0.05 versus control group. ^†^ *P* < 0.05 versus NASH group. 

### 3.3. Biochemical Parameters

Plasma transaminases (ALT, AST) were not significantly affected by any of the treatments (Table 2). The level of LDL rose significantly in the NASH group as compared with the control group (*P* < 0.05). The N15 treatment reduced significantly this parameter (*P* < 0.05). Triglycerides were lowered by NASH induction as compared to the control group and none of the treatments was able to correct this parameter. In contrast, serum LDH was highly increased by NASH treatment as compared to the control group (*P* < 0.05) and N30 as well as N15 treatments succeeded in returning this parameter to control values, in a very close manner to the UDCA treatment (*P* < 0.05) (Table 2). 

Measurements were obtained from plasma of rats in the fasting state, after 12 weeks of standard diet (Control) or high-fat diet alone (NASH), or with treatment for the last 6 weeks with UDCA (17.2 mg/kg), NCX 1000 (30 or 15 mg/kg) (N30, N15 resp.), or NCX 1000 plus vitamin E (15 mg/kg and 100 mg/kg, respe.) (N15 + VitE). AST: aspartate aminotransferase; ALT: alanine aminotransferase; TC: total cholesterol; HDL: high density lipoprotein cholesterol; LDL: low density lipoprotein cholesterol; TG: triglycerides; LDH: lactate dehydrogenase. Values are expressed as means ± SEM of 6–10 rats. The *P* values for the ANOVA Fisher's PLSD test are given. For pairwise comparisons *P < 0.05 versus control group and ^†^ *P* < 0.05 versus NASH group.

### 3.4. Histological Evaluation

**Figure 2:**
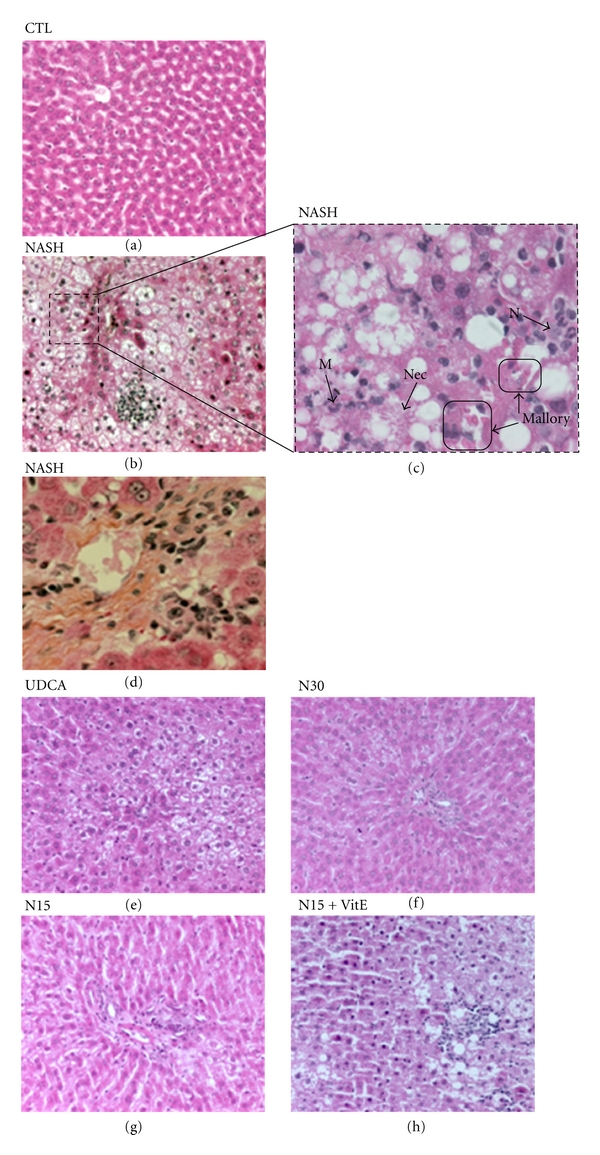
Effect of treatments on liver histology.

Liver histology is currently the gold standard test for the diagnosis of NASH. HPS staining of liver sections from rats in the control group (Figure 2(a)) showed normal morphological features. The tissues of all animals from the NASH group (Figure 2(b)) were found to exhibit macrovesicular steatosis of grade 2 or 3 confirming that more than a third of hepatocytes contained macrovesicular fat (Table 3; *P* < 0.05 Chi square test). Moreover, Mallory bodies (Figure 2(c)) and collagen deposition (Figure 2(d)) were encountered exclusively in a number of samples from the NASH group. All treatments yielded statistically significant improvements in histology. As compared to NASH animals, 60 to 70% of rats in groups treated with N30, N15, or NCX 1000 combined with vitamin E had less than a third of hepatocytes containing macrovesicular fat, in the same way as the UDCA treated rats (Figures 2(e) to 2(h); Table 3, *P* < 0.05 Chi square test). The infiltration of inflammatory cells in hepatic tissue was very seldom seen in control animals. In contrast, 100% of NASH animals suffered from moderate to severe inflammatory cell infiltration, a significant change from control animals (Table 3, *P* < 0.05 Chi square test). N30 treatment significantly improved this parameter, similarly to UDCA treatment (Table 3, *P* < 0.05 Chi square test). Reducing the dose of NCX 1000 with or without combination with vitamin E also yielded a slightly inferior yet significant beneficial effect. 

Histological scoring for steatosis and inflammation from rats fed a standard liquid diet for 12 weeks (Control) or a high-fat liquid diet alone (NASH), or with treatment for the last 6 weeks with UDCA (17.2 mg/kg), NCX 1000 (30 or 15 mg/kg) (N30, N15 resp.), or NCX 1000 plus vitamin E (15 mg/kg and 100 mg/kg, resp.) (N15 + VitE). Macrovesicular steatosis grade 0: absent; grade 1: less than 33%; grade 2: 33–66%; grade 3: more than 66%. Inflammation 0: normal; 1: mild; 2: moderate; 3: severe. 

Representative HPS staining of rat liver sections. The control (A-CTL, 20X**)** group received 12 weeks of standard liquid diet and showed normal histology. The NASH (B, C, D-NASH, 20X, and 63X) group received a high-fat liquid diet during 12 weeks and revealed macrovesicles of fat, hepatocyte ballooning, inflammatory cells infiltration such as monocytes (M) and neutrophils (N), necrosis (Nec), and Mallory bodies (Mallory). 12 weeks of high-fat liquid diet with treatment during the last 6 weeks with either UDCA 17.2 mg/kg (E-UDCA, 20X), NCX 1000 30 or 15 mg/kg (F-N30 and G-N15, resp; 20X), or NCX 1000 15 mg/kg plus vitamin E 100 mg/kg (H-N15 + VitE, 20X), displayed less macrovesicular steatosis and inflammation than the NASH group.

### 3.5. Inflammatory Cytokines and Adipokines

**Figure 3:**
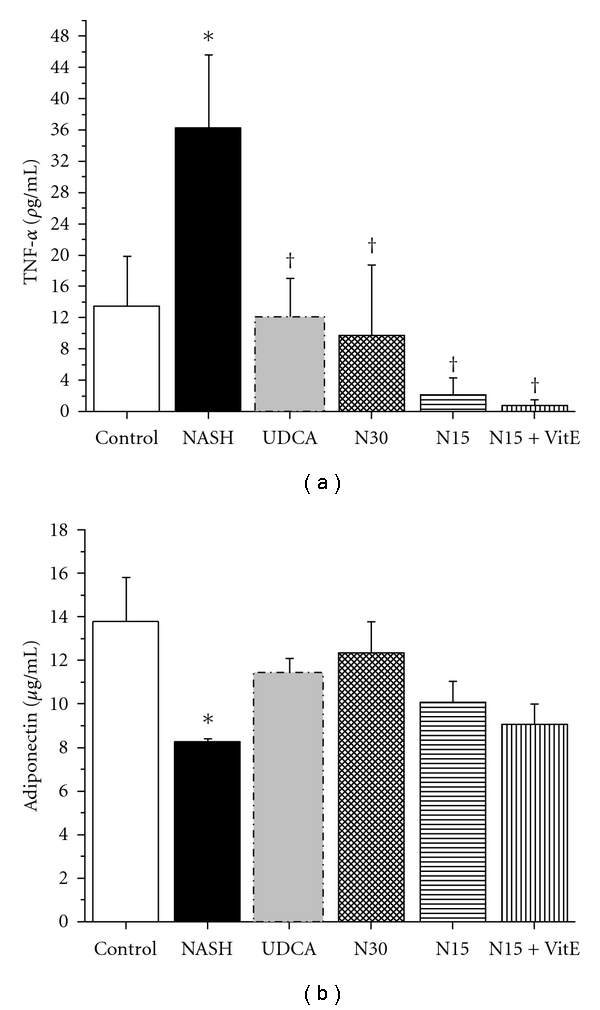
Effect of treatments on inflammatory cytokines and adipokines.

TNF-*α* is an inflammatory cytokine produced by adipose tissue and macrophages (including liver Kupffer cells). It is known to impair insulin signaling, leading eventually to insulin resistance. In the NASH group, plasma TNF-*α* was higher than in the control group (Figure 3(a); 36.3 ± 9.3 versus 13.5 ± 6.4 *ρ*g/mL, resp.; *P* < 0.05). All drug treatments diminished circulating TNF-*α* level significantly, in a similar and dose-dependent manner (N30, N15) or more effective manner (N15 + VitE) than UDCA, as compared to the NASH group (*P* < 0.05). Adiponectin is a hormone secreted by adipose tissue that is known to decrease during fatty liver disease and the metabolic syndrome. Indeed, the high-fat NASH treatment induced a significant reduction in serum adiponectin as compared to the control group (Figure 3(b); *P* < 0.05). All drug treatments, notably the N30 group, had a tendency to increase circulating adiponectin but this effect failed to reach statistical analysis.

TNF-*α* Figure 3(a) and adiponectin Figure 3(b) were measured using ELISA kits in plasma and serum, respectively, and were obtained from rats in the fasting state, after 12 weeks of standard liquid diet (Control), high-fat liquid diet alone (NASH), or with treatment for the last 6 weeks with UDCA (17.2 mg/kg), NCX 1000 (30 or 15 mg/kg) (N30, N15 resp.), or NCX 1000 plus vitamin E (15 mg/kg + 100 mg/kg) (N15 + VitE). Values are expressed as means ± SEM of 6–10 rats. *P < 0.05 versus control group. ^†^ *P* < 0.05 versus NASH group.

### 3.6. Oxidative Stress

**Figure 4:**
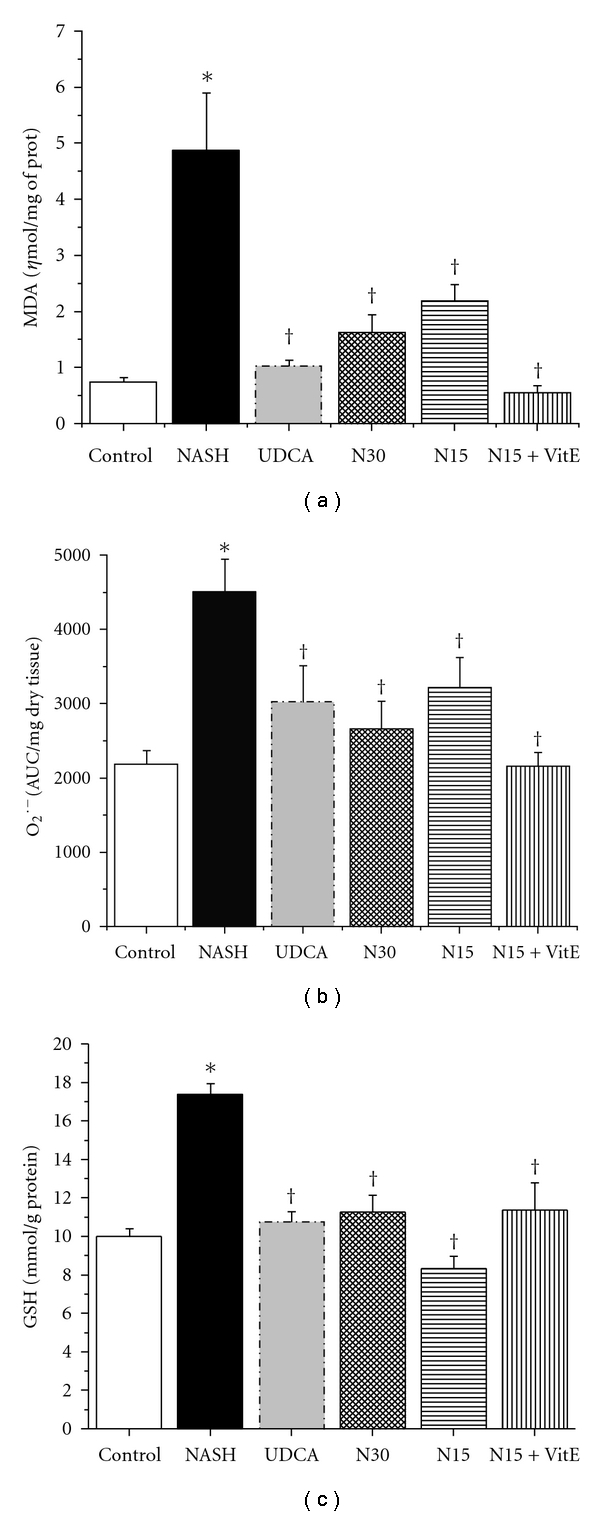
Effect of treatments on oxidative stress.

Twelve weeks of the NASH-inducing high-fat diet caused hepatic malondialdehyde (MDA) levels, a product of lipid peroxidation, to more than quadruple as compared to the control group (4.87 ± 1.03 versus 0.74 ± 0.08 *η*mol/mg of protein, resp; *P* < 0.05, Figure 4(a)). N30 treatment during the last six weeks normalized liver MDA levels in an equivalent way to UDCA but in a better way than N15, suggesting a dose-effect relationship. When vitamin E was combined with N15, MDA levels decreased to levels slightly below those of the control group, suggesting a possible additive effect. 

The superoxide anion (O_2_^∙−^), an important reactive oxygen species (ROS), is produced by the liver during oxidative stress.  Figure 4(b) shows that the level of hepatic O_2_^∙−^ exhibited a near twofold increase in the NASH group as compared to the control group (4,506 ± 440 and 2,186 ± 183 AUC/mg dry tissue, resp.; *P* < 0.05). All treatments, notably N30 and N15  + VitE, decreased O_2_^∙−^ levels significantly, to values similar to that of UDCA reference group. 

Hepatic-reduced glutathione (GSH) is a potent antioxidant produced by the liver as a mechanism of intracellular defense. As illustrated by Figure 4(c), GSH level was significantly elevated in the NASH group when compared with the control group (17.4 ± 0.6 versus 10.0 ± 0.4 mmol/g of protein, *P* < 0.05). These results are demonstrative of the stress induced by a 12-week high-fat liquid diet. Again, N30, N15 + VitE, and N15 treatments, in order of importance, returned hepatic glutathione to levels similar to that of the control and UDCA groups (*P* < 0.05).

Hepatic malondialdehyde (MDA) Figure 4(a),  O_2_^∙−^ production Figure 4(b), and GSH concentration Figure 4(c) were measured in fresh or frozen liver tissue obtained from rats in the fasting state, after 12 weeks of standard liquid diet (Control), high-fat liquid diet alone (NASH), or with treatment for the last 6 weeks with UDCA (17.2 mg/kg), NCX 1000 (30 or 15 mg/kg) (N30, N15 resp.), or NCX 1000 plus vitamin E (15 mg/kg + 100 mg/kg) (N15 + VitE). Values are expressed as means ± SEM of 6–10 rats. *P < 0.05 versus control group. ^†^ *P* < 0.05 versus NASH group.

### 3.7. Mitochondrial ATP

**Figure 5:**
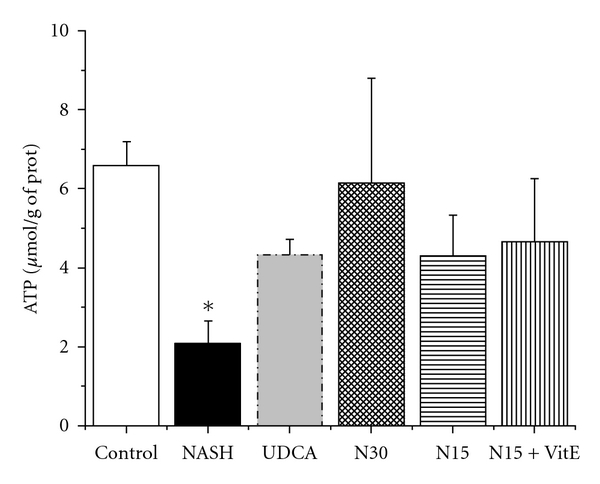
Effect of treatments on mitochondrial ATP.

As shown in Figure 5, hepatic mitochondrial ATP level was lower in the NASH group than in the control group (*P* < 0.05). This is indicative of cellular energetic imbalance caused by a 12-week high-fat liquid diet. All drug treatment groups yielded ATP values that were statistically similar to both the UDCA and the control groups.

ATP was measured in freshly isolated mitochondria obtained from rats in the fasting state, after 12 weeks of standard liquid diet (Control), high-fat liquid diet alone (NASH), or with treatment for the last 6 weeks with UDCA (17.2 mg/kg), NCX 1000 (30 or 15 mg/kg) (N30, N15 resp.), or NCX 1000 plus vitamin E (15 mg/kg + 100 mg/kg) (N15 + VitE). Values are expressed as means ± SEM of 6–10 rats. Overall ANOVA was not significant among all groups. Nonetheless, single *t*-test revealed significance for NASH versus control group *P < 0.05. 

### 3.8. Expression of CYP2E1 and PPAR-*α*

**Figure 6:**
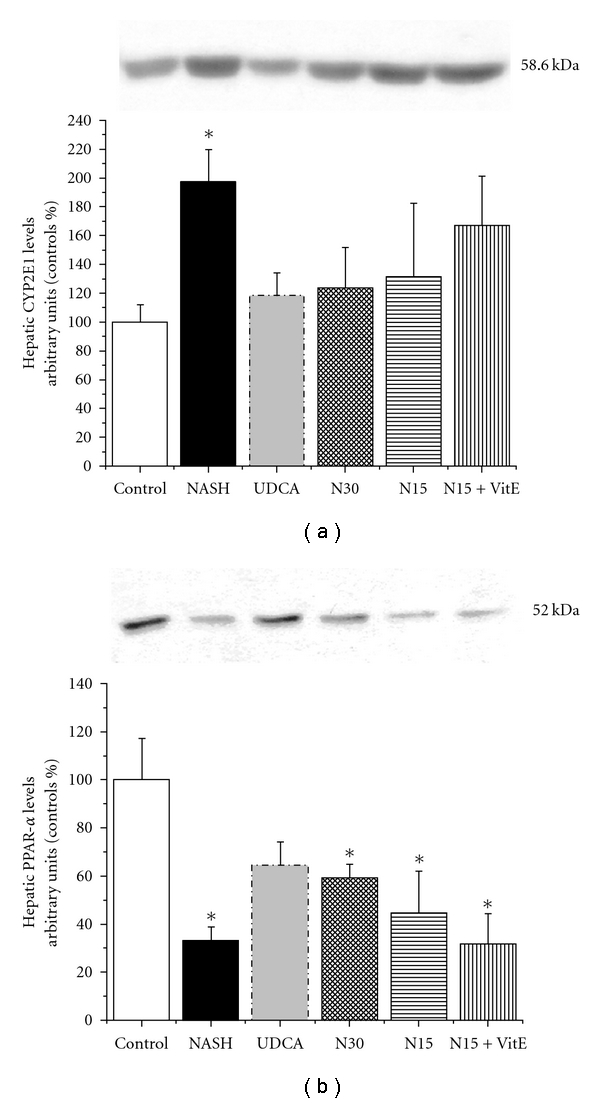
Effect of treatments on CYP2E1 and PPAR-*α*.

The expression of the metabolizing enzyme cytochrome P450 CYP2E1 is upregulated by the overproduction of ketones, which is due to impaired mitochondrial *β*-oxidation as well as increased peroxisomal *β*-oxidation and microsomal *ω*-oxidation of free fatty acids (FFAs) in hepatocytes. Western blots showed that the microsomal expression of cytochrome P450 CYP2E1 was increased after 12 weeks of high-fat liquid diet in livers from the NASH group (98% above control, *P* < 0.05). CYP2E1 expression in the drug treatment groups, except in that of N15 + VitE, was lowered to values close to those of the control group, but this difference was not statistically significant from the NASH group (Figure 6(a)). 

The expression of peroxisome proliferator-activated receptor-*α* (PPAR-*α*), a transcription factor responsible for the activation of fat metabolism genes, was significantly decreased in the livers of NASH animals as compared to values observed in the control group (33% of control, *P* < 0.05), but was not affected significantly by any treatment in our study when compared to the NASH group as shown by the representative western blots (Figure 6(b)).

Representative western blots were performed on liver microsomal fraction for CYP2E1 Figure 6(a) or whole liver homogenates for PPAR-*α* Figure 6(b) to reveal their protein expression. Livers were obtained from rats in the fasting state, after 12 weeks of standard liquid diet (CTL), high-fat liquid diet alone (NASH), or with treatment for the last 6 weeks with UDCA (17.2 mg/kg), NCX 1000 (30 or 15 mg/kg) (N30, N15 resp.), or NCX 1000 plus vitamin E (15 mg/kg + 100 mg/kg) (N15 + VitE). Values are expressed as means ± SEM of 4 samples. *P < 0.05 versus control group. 

## 4. Discussion

NASH is a threatening liver disease that can progress through fibrosis and cirrhosis. It has been associated with the metabolic syndrome and its incidence is thus following a similar expanding trend, reaching 3% of worldwide populations [2, 41]. At present, no efficient remedy exists. Treatment is still centered on major lifestyle changes (weight loss, healthy eating, and exercise) [42], with emerging therapeutic avenues focused on improving insulin resistance, reducing inflammation as well as oxidative stress [43, 44]. In the search for potential novel and beneficial treatment options, NCX 1000 has attracted our attention. Indeed, this substance exerts a wide spectrum of beneficial effects in the liver [25–30]. We therefore evaluated the beneficial effect of NCX 1000, combined or not to vitamin E, in an experimental *in vivo *rat model of NASH and used UDCA, its parent molecule, as a reference treatment.

We selected a NASH rat model developed by Lieber and colleagues, that is based on the consumption of a high-fat liquid diet [32]. Our results confirmed that a 12-week treatment with a high-fat liquid diet triggered all the salient features of NASH in rats and reproduced the majority of the clinical expressions of the human disease. Furthermore, in preliminary studies, the cardinal histological features of NASH (steatosis, inflammation and fibrosis) were already present after six weeks of the Lieber diet, in accordance with the original description of the model [32]. We therefore began treatments at that time point, with the intention of treating the disease.

Our results showed that N30 treatment for six weeks was very efficient at reversing the development of NASH, despite the continued intake of the high-fat liquid diet. Indeed, the cardinal features of NASH were effectively antagonized. Firstly, the histological grade of steatosis was dramatically improved. NCX 1000 treatment at a dose of 30 mg/kg was also efficient at correcting NASH-associated inflammation, as seen by the significant histological improvement in immune cell infiltration and by the reduction in circulating TNF-*α*. Histological evidence of necrosis or fibrosis was also not observed in the livers of N30 animals, as opposed to NASH congeners. Finally, N30 treatment successfully normalized all measured parameters of hepatic oxidative stress (lipid peroxidation, ROS production, and glutathione content). This was consistent with the return of circulating LDH towards normal values observed in control animals. Such antioxidant, cytoprotective, and antifibrogenic effects of NCX 1000 are in line with previous reports [26, 29], including our own studies using an amiodarone-induced NASH-like *in vitro* model [31]. In addition, microsomal CYP2E1 expression tended to decrease in NCX 1000 treated animals, in line with the improvement of oxidative stress, but such normalization failed to reach statistical significance. A correlation was recently established between the expression of other members of the cytochrome P450 enzymes, TNF-*α*, and the progression of NAFLD [45]. Further investigation will be necessary to assess the effects of our treatments on these other isoforms of cytochrome P450.

NCX 1000 also restored several metabolic parameters associated with NASH. This was notably the case with indices of insulin resistance, as attested by the normalization of insulinemia and of HOMA-IR. This is not surprising given the known relation between liver steatosis and the development of insulin resistance [10]; the reduction in steatosis induced by NCX 1000 thus contributing to improve insulin sensitivity. Together with the decrease in plasma TNF-*α* already mentioned, the tendency for serum adiponectin levels to rise back toward normal control values may also have contributed in restoring insulin sensitivity in N30 animals [5, 19, 46, 47]. However, perturbations in blood lipids, notably increased LDL and decreased PPAR-*α* expression, observed in NASH animals were not corrected by N30 treatment. 

The treatment with a lower dose of NCX 1000 (N15) seemed to be less efficient than N30, notably in terms of LDH release, MDA, and O_2_^∙−^ production, suggesting a dose-dependent effect more specifically at the level of oxidative stress parameters. Indeed, for all other parameters, N15 had very similar effects to those of N30. 

We also assessed the combination of NCX 1000 with natural antioxidants, in a rationale to diminish the dose of the chemical drug, hence to reduce the risk of possible adverse reactions. Vitamin E (*α*-tocopherol) is a lipophilic antioxidant widely known for its beneficial effects on NAFLD. In fact, it reduces lipid peroxidation, improves fibrosis, attenuates inflammation, and stabilizes membranes due to its lipid solubility [48, 49]. It has already been combined with UDCA in a small-scale clinical study for the treatment of NASH and improved liver steatosis and transaminase levels as compared to UDCA with placebo [19, 22]. We also recently showed that vitamin E, as opposed to hydrophilic antioxidants, significantly enhanced the antioxidant and cytoprotective potential of NCX 1000 in cultured hepatocytes with NASH-like lesions [31]. Results from the present study show that the association of vitamin E with N15 significantly enhanced the beneficial effects of NCX 1000 against NASH-induced lesions. This was especially true at the level of lipid peroxidation and superoxide anion production. Thus, it appears that vitamin E can offer an additional antioxidant protection that may synergize with lower doses of NCX 1000. This is consistent with the concept that vitamin E's main target in experimental NASH is oxidative stress. 

Overall, our results thus clearly demonstrate that NCX 1000 holds a promising potential for the treatment of NASH and suggest that the dose can be lowered if the drug is combined with vitamin E. Further studies will be needed to characterize precisely the ratio between NCX 1000 and vitamin E needed to obtain the maximum effect with the lower doses.

Finally, NCX 1000 exerted antioxidant, anti-inflammatory, cytoprotective, and insulin sensitizing activities as efficient as UDCA, at a clinically relevant dose of 17.2 mg/kg. In the liver, NCX 1000 is expected to be metabolized to release NO and an equimolar amount of UDCA. The higher N30 dose used in this study should therefore theoretically have provided an equivalent amount to the UDCA dose tested. Since the effects of NCX 1000 and UDCA were comparable, our study would argue for a minimal role of NO in the observed beneficial action in NASH. This conclusion differs from our previous *in vitro* study [31], where equimolar NCX 1000 afforded better protection than UDCA to mouse hepatocytes treated with amiodarone to produce NASH-like injuries. Further studies will thus be necessary to fully assess the role of the NO in the current model. NCX 1000 at a lower dose, combined with vitamin E, did afford an advantage by reducing the corresponding dose of UDCA required. Future studies should therefore also compare NCX 1000 and UDCA, each combined with vitamin E to fully assess if NCX 1000 offers any advantage over the parent compound.

In conclusion, our study demonstrates for the first time that a therapeutic treatment with NCX 1000 is effective in reducing steatosis, inflammation, oxidative stress, and insulin resistance in an* in vivo* rat model of diet-induced NASH. In this model, NCX 1000 does not appear to offer any advantage over equimolar amounts of the parent compound UDCA. However, the results of our study also support the use of combinations of lower doses of NCX 1000 with natural lipophilic antioxidants, such as vitamin E, as a valid option for the treatment of NASH. A clinical validation of this approach will remain mandatory.

##  Conflict of Interests

Dr. Jean Spénard is employed by Axcan Pharma Inc. that sponsored the study. He does not stand to gain personally from the publication of this paper.

## Figures and Tables

**Table 1:** Effect of treatments on body and liver weight.

Parameters	BW (g)	WG (g)	LW (g)	LI (%)
Control	563 ± 19	475 ± 8	14.4 ± 0.5	2.5 ± 0.1
NASH	523 ± 17	464 ± 15	16.2 ± 0.7	3.1 ± 0.1*
UDCA	505 ± 15	436 ± 15	13.4 ± 0.6^†^	2.7 ± 0.1^†^
N30	537 ± 26	461 ± 25	14.0 ± 0.9^†^	2.6 ± 0.1^†^
N15	554 ± 16	478 ± 17	14.6 ± 0.7	2.6 ± 0.1^†^
N15 + VitE	547 ± 12	469 ± 14	15.0 ± 0.7	2.7 ± 0.1^†^

ANOVA	0.292	0.360	0.248	0.021

**Table 2:** Effect of treatments on biochemical and lipid parameters.

Parameters	AST	ALT	TC	HDL	LDL	TG	LDH
	U/L	U/L	mmol/L	mmol/L	mmol/L	mmol/L	U/L
Control	53.5 ± 4.9	30.2 ± 3.6	0.75 ± 0.07	0.37 ± 0.03	0.09 ± 0.04	0.72 ± 0.02	62 ± 5
NASH	67.8 ± 9.5	39.8 ± 5.6	0.82 ± 0.02	0.35 ± 0.05	0.25 ± 0.03*	0.31 ± 0.04*	111 ± 13*
UDCA	59.2 ± 2.5	34.2 ± 2.4	0.54 ± 0.03^†^	0.27 ± 0.01	0.17 ± 0.02	0.29 ± 0.03*	68 ± 4^†^
N30	54.2 ± 5.8	31.7 ± 4.5	0.75 ± 0.06	0.38 ± 0.03	0.21 ± 0.03	0.35 ± 0.03*	68 ± 8^†^
N15	57.9 ± 5.7	36.5 ± 4.9	0.64 ± 0.05	0.32 ± 0.03	0.14 ± 0.02^†^	0.31 ± 0.02*	77 ± 12^†^
N15 + VitE	51.7 ± 3.9	29.1 ± 2.7	0.70 ± 0.06	0.29 ± 0.03	0.23 ± 0.04	0.36 ± 0.03*	80 ± 13

ANOVA	0.448	0.461	0.033	0.053	0.012	<0.0001	0.077

**Table 3:** Liver histology scoring for macrovesicular steatosis and inflammatory cells infiltration.

Groups	*n*		Steatosis					Inflammation			
			0	1	2	3		0	1	2	3
Control	6		6	0	0	0		6	0	0	0
NASH	6		0	0	2	4		0	0	4	2
UDCA	10		3	3	3	1		3	4	3	0
N30	10		2	5	2	1		3	4	3	0
N15	10		5	2	1	2		3	4	2	1
N15 + VitE	10		3	3	2	2		4	3	2	1

Chi square			*P* < 0.05					*P* < 0.05			

## References

[1] Ludwig J, Viggiano TR, McGill DB, Ott BJ (1980). Nonalcoholic steatohepatitis: mayo Clinic experiences with a hitherto unnamed disease. *Mayo Clinic Proceedings*.

[2] Bellentani S, Marino M (2009). Epidemiology and natural history of non-alcoholic fatty liver disease (NAFLD). *Annals of Hepatology*.

[3] Marchesini G, Bugianesi E, Forlani G (2003). Nonalcoholic fatty liver, steatohepatitis, and the metabolic syndrome. *Hepatology*.

[4] Pagano G, Pacini G, Musso G (2002). Nonalcoholic steatohepatitis, insulin resistance, and metabolic syndrome: further evidence for an etiologic association. *Hepatology*.

[5] Capeau J (2008). Insulin resistance and steatosis in humans. *Diabetes &amp; Metabolism*.

[6] Malhi H, Gores GJ (2008). Molecular mechanisms of lipotoxicity in nonalcoholic fatty liver disease. *Seminars in Liver Disease*.

[7] Brunt EM, Tiniakos DG (2005). Pathological features of NASH. *Frontiers in Bioscience*.

[8] Nonomura A, Enomoto Y, Takeda M (2005). Clinical and pathological features of non-alcoholic steatohepatitis. *Hepatology Research*.

[9] Malhi H, Gores GJ (2008). Cellular and molecular mechanisms of liver injury. *Gastroenterology*.

[10] Byrne CD, Olufad R, Bruce KD, Cagampang FR, Ahmed MH (2009). Metabolic disturbances in non-alcoholic fatty liver disease. *Clinical Science*.

[11] Neuschwander-Tetri BA, Caldwell SH (2003). Nonalcoholic steatohepatitis: summary of an AASLD single topic conference. *Hepatology*.

[12] Ljubuncic P, Tanne Z, Bomzon A (2000). . Ursodeoxycholic acid suppresses extent of lipid peroxidation in diseased liver in experimental cholestatic liver disease. *Digestive Diseases and Sciences*.

[13] Buko VU, Lukivskaya OYA, Zavodnik LB, Sadovnichy VV, Petushok NE, Tauschel HD (2002). Antioxidative effect of ursodeoxycholic acid in the liver of rats with oxidative stress caused by gamma-irradiation. *Ukrainskii Biokhimicheskii Zhurnal*.

[14] Chang CY, Argo CK, Al-Osaimi AMS, Caldwell SH (2006). Therapy of NAFLD: antioxidants and cytoprotective agents. *Journal of Clinical Gastroenterology*.

[15] Angulo P (2002). Use of ursodeoxycholic acid in patients with liver disease. *Current Gastroenterology Reports*.

[16] Festi D, Montagnani M, Azzaroli F (2007). Clinical efficacy and effectiveness of ursodeoxycholic acid in cholestatic liver diseases. *Current Clinical Pharmacology*.

[17] Trauner M, Fickert P, Halilbasic E, Moustafa T (2008). Lessons from the toxic bile concept for the pathogenesis and treatment of cholestatic liver diseases. *Wiener Medizinische Wochenschrift*.

[18] Kumar D, Tandon RK (2001). Use of ursodeoxycholic acid in liver diseases. *Journal of Gastroenterology and Hepatology*.

[19] Balmer ML, Siegrist K, Zimmermann A, Dufour JF (2009). Effects of ursodeoxycholic acid in combination with vitamin E on adipokines and apoptosis in patients with nonalcoholic steatohepatitis. *Liver International*.

[20] Laurin J, Lindor KD, Crippin JS (1996). Ursodeoxycholic acid or clofibrate in the treatment of non-alcohol-induced steatohepatitis: a pilot study. *Hepatology*.

[21] Georgescu EF, Georgescu M (2007). Therapeutic options in non-alcoholic steatohepatitis (NASH). Are all agents alike? Results of a preliminary study. *Journal of Gastrointestinal and Liver Diseases*.

[22] Dufour J, Oneta CM, Gonvers JJ (2006). Randomized placebo-controlled trial of ursodeoxycholic acid with vitamin e in nonalcoholic steatohepatitis. *Clinical Gastroenterology and Hepatology*.

[23] Fan JG, Zhong L, Tia LIY, Xu ZJ, Li MS, Wang GL (2005). Effects of ursodeoxycholic acid and/or low-calorie diet on steatohepatitis in rats with obesity and hyperlipidemia. *World Journal of Gastroenterology*.

[24] Lindor KD, Kowdley KV, Heathcote EJ (2004). Ursodeoxycholic acid for treatment of nonalcoholic steatohepatitis: results of a randomized trial. *Hepatology*.

[25] Fiorucci S, Antonelli E, Brancaleone V (2003). NCX-1000, a nitric oxide-releasing derivative of ursodeoxycholic acid, ameliorates portal hypertension and lowers norepinephrine-induced intrahepatic resistance in the isolated and perfused rat liver. *Journal of Hepatology*.

[26] Fiorucci S, Antonelli E, Distrutti E (2004). Liver delivery of NO by NCX-1000 protects against acute liver failure and mitochondrial dysfunction induced by APAP in mice. *British Journal of Pharmacology*.

[27] Fiorucci S, Antonelli E, Morelli A (2003). Nitric oxide and portal hypertension: a nitric oxide-releasing derivative of ursodeoxycholic acid that selectively releases nitric oxide in the liver. *Digestive and Liver Disease*.

[28] Fiorucci S, Antonelli E, Morelli O (2001). NCX-1000, a NO-releasing derivative of ursodeoxycholic acid, selectively delivers NO to the liver and protects against development of portal hypertension. *Proceedings of the National Academy of Sciences of the United States of America*.

[29] Fiorucci S, Mencarelli A, Palazzetti B, Del Soldato P, Morelli A, Ignarro LJ (2001). An NO derivative of ursodeoxycholic acid protects against Fas-mediated liver injury by inhibiting caspase activity. *Proceedings of the National Academy of Sciences of the United States of America*.

[30] Loureiro-Silva MR, Cadelina GW, Iwakiri Y, Groszmann RJ (2003). A liver-specific nitric oxide donor improves the intra-hepatic vascular response to both portal blood flow increase and methoxamine in cirrhotic rats. *Journal of Hepatology*.

[31] Ouazzani-Chahdi A, Elimadi A, Chabli A, Spénard J, Colin P, Haddad PS (2007). Combining ursodeoxycholic acid or its NO-releasing derivative NCX-1000 with lipophilic antioxidants better protects mouse hepatocytes against amiodarone toxicity. *Canadian Journal of Physiology and Pharmacology*.

[32] Lieber CS, Leo MA, Mak KIM (2004). Model of nonalcoholic steatohepatitis. *American Journal of Clinical Nutrition*.

[33] Huet PM, Nagaoka MR, Desbiens G (2004). Sinusoidal endothelial cell and hepatocyte death following cold ischemia-warm reperfusion of the rat liver. *Hepatology*.

[34] Kleiner DE, Brunt EM, Van Natta M (2005). Design and validation of a histological scoring system for nonalcoholic fatty liver disease. *Hepatology*.

[35] Brunt EM, Janney CG, Di Bisceglie AM, Neuschwander-Tetri BA, Bacon BR (1999). Nonalcoholic steatohepatitis: a proposal for grading and staging the histological lesions. *American Journal of Gastroenterology*.

[36] Hettihawa LMPS, Jayasinhe SS, Gunasekara SW, Weerarathna TP (2006). Comaparison of insulin resistance by indirect methods—HOMA, QUICKI, and MCAuley—with fasting insulin in patients with type 2 diabetes in Galle, Sri Lanka: a pilot study. *Online Journal of Health and Allied Sciences*.

[37] Johnson DLH, Lardy H, Estabrook RW, Pullman ME (1967). Isolation of liver and kidney mitochondria. *Methods in Enzymology*.

[38] Lowry OH, Rosebrough NJ, Farr AL, Randall RJ (1951). Protein measurement with the Folin phenol reagent. *The Journal of Biological Chemistry*.

[39] Ligeret H, Brault A, Vallerand D, Haddad Y, Haddad PS (2007). Antioxidant and mitochondrial protective effects of silibinin in cold preservation-warm reperfusion liver injury. *Journal of Ethnopharmacology*.

[40] Oliveira PJ, Rolo AP, Palmeira CM, Moreno AJM (2001). Carvedilol reduces mitochondrial damage induced by hypoxanthine/xanthine oxidase: relevance to hypoxia/reoxygenation injury. *Cardiovascular Toxicology*.

[41] Farrell GC, Larter CZ (2006). Nonalcoholic fatty liver disease: from steatosis to cirrhosis. *Hepatology*.

[42] Caldwell S, Lazo M (2009). Is exercise an effective treatment for NASH? Knowns and unknowns. *Annals of Hepatology*.

[43] Ali R, Cusi K (2009). New diagnostic and treatment approaches in non-alcoholic fatty liver disease (NAFLD). *Annals of Medicine*.

[44] Lam BP, Younossi ZM (2009). Treatment regimens for non-alcoholic fatty liver disease. *Annals of Hepatology*.

[45] Fisher CD, Lickteig AJ, Augustine LM (2009). Hepatic cytochrome P450 enzyme alterations in humans with progressive stages of non-alcoholic fatty liver disease. *Drug Metabolism and Disposition*.

[46] Hui JM, Hodge A, Farrell GC, Kench JG, Kriketos A, George J (2004). Beyond insulin resistance in NASH: TNF-*α* or adiponectin?. *Hepatology*.

[47] Larter CZ, Farrell GC (2006). Insulin resistance, adiponectin, cytokines in NASH: which is the best target to treat?. *Journal of Hepatology*.

[48] Hasegawa T, Yoneda M, Nakamura K, Makino I, Terano A (2001). Plasma transforming growth factor-*β*1 level and efficacy of *α*-tocopherol in patients with non-alcoholic steatohepatitis: a pilot study. *Alimentary Pharmacology and Therapeutics*.

[49] Lavine JE (2000). Vitamin E treatment of nonalcoholic steatohepatitis in children: a pilot study. *Journal of Pediatrics*.

